# Nano-FTIR spectroscopic identification of prebiotic carbonyl compounds in Dominion Range 08006 carbonaceous chondrite

**DOI:** 10.1038/s41598-021-91200-8

**Published:** 2021-06-02

**Authors:** Mehmet Yesiltas, Timothy D. Glotch, Bogdan Sava

**Affiliations:** 1 Faculty of Aeronautics and Space Sciences, Kirklareli University, Kirklareli, Turkey; 2 Department of Geosciences, Stony Brook University, Stony Brook, NY USA; 3 Neaspec GmbH, 85540 Haar, Munich, Germany

**Keywords:** Meteoritics, Astrobiology, Characterization and analytical techniques

## Abstract

Meteorites contain organic matter that may have contributed to the origin of life on Earth. Carbonyl compounds such as aldehydes and carboxylic acids, which occur in meteorites, may be precursors of biologically necessary organic materials in the solar system. Therefore, such organic matter is of astrobiological importance and their detection and characterization can contribute to the understanding of the early solar system as well as the origin of life. Most organic matter is typically sub-micrometer in size, and organic nanoglobules are even smaller (50–300 nm). Novel analytical techniques with nanoscale spatial resolution are required to detect and characterize organic matter within extraterrestrial materials. Most techniques require powdered samples, consume the material, and lose petrographic context of organics. Here, we report the detection of nanoglobular aldehyde and carboxylic acids in a highly primitive carbonaceous chondrite (DOM 08006) with ~ 20 nm spatial resolution using nano-FTIR spectroscopy. Such organic matter is found within the matrix of DOM 08006 and is typically 50–300 nm in size. We also show petrographic context and nanoscale morphologic/topographic features of the organic matter. Our results indicate that prebiotic carbonyl nanoglobules can form in a less aqueous and relatively elevated temperature-environment (220–230 °C) in a carbonaceous parent body.

## Introduction

Carbonaceous chondrites are the most primitive meteorite samples in the solar system, and they retain records of their formation and post-accretionary processes. They also contain a wide range of extraterrestrial organic compounds that can provide information about the processes in the interstellar medium and early solar system. Meteoritic organics include aliphatic and aromatic hydrocarbons, amino acids, carboxylic acids, sulfonic and phosphonic acids, alcohols, aldehydes, ketones, sugars, amines, amides, nitrogen, and sulfur heterocycles^1^. These organic molecules may have formed prior to accretion in the interstellar medium or solar nebula. Alternatively, they may have formed in situ in the parent bodies through a variety of mechanisms. Regardless of their origin, they are affected by a variety of secondary processes, which complicates the understanding of the early solar system processes that formed them. Aqueous alteration and thermal metamorphism are the most dominant processes that not only affect mineralogy but also the organic content of meteorites. For instance, pervasive aqueous alteration of the primary phases such as anhydrous silicates can cause the formation of secondary phases such as phyllosilicates, carbonates, and sulfates^2^. Thermal metamorphism, which likely occurred after aqueous alteration, can cause the dehydration of matrix phyllosilicates, decomposition of carbonates, and recrystallization of other phases^3^.

To better understand the earliest geologic processes in the Solar System, we must interrogate the most primitive and the least altered samples that are available. CO chondrites exhibit the complete metamorphic sequence from type 3.0 to 3.9 due to varying amounts of thermal metamorphism^4,5^; however, hydrothermal alteration appears to have altered the mineralogy and chemistry of the higher CO petrographic types^6^. As such, like other well-established carbonaceous chondrite groups, the properties of members of the CO3 chondrites are attributed to the thermal metamorphic processes they have been subjected to. In this context, the subtype CO3.0 chondrites can be considered to be the least altered and relatively more primitive.

DOM 08006 is a particularly special sample. It is a subtype 3.0 CO chondrite, indicating that it has undergone little aqueous and thermal alteration^7^. It is one of the most primitive carbonaceous chondrites, and the most primitive CO chondrite known to date^7^. This claim is supported by several pieces of evidence. For instance, presolar silicate grains are extremely sensitive to aqueous alteration and thermal metamorphism^8^. DOM 08006 contains a higher proportion of presolar grains than any other chondrite, with abundances comparable to interplanetary dust particles^9,10^. Additionally, DOM 08006 contains a low amount of straight-chain amino acids, which is lower than the most primitive CO chondrite ALH 77307^11^. The fact that such amino acids are produced during thermal metamorphism indicates that DOM 08006 may have undergone very little, if any, thermal metamorphism^7^. Thus, DOM 08006 is an extremely important meteorite for providing valuable insights regarding the primitive unaltered nature of nebular materials that accreted to planetesimals in the protoplanetary disk, as well for understanding the formation/evolution conditions of the early solar system^10^.

Meteoritic organic matter is typically sub-micron in size^12,13^. Some of those, such as organic nanoglobules^14,15^, are even smaller (50–500 nm). Evidently, novel analytical techniques with nanoscale spatial resolution are required to detect and characterize such organic matter within meteorites. It is also important to preserve and not alter the organic matter and the petrographic context in any way during the investigation. Electron and X-ray microscopy (TEM, STXM) can induce radiation damage on beam-sensitive organic materials^16^, potentially altering the organic matter permanently. They also require samples to be coated, introducing an additional possibility for contamination. Gas chromatography-mass spectroscopy (GCMS) requires powdered samples, as such the petrographic context of the sample is permanently lost, and the sample is completely consumed. Fourier transform infrared (FTIR) spectroscopy is a completely non-destructive analytical technique and has been utilized to characterize organic and inorganic constituents of a variety of extraterrestrial samples including meteorites^17–25^. However, its spatial resolution is not high enough to detect and identify spectral signatures of organic molecules that are smaller than a micrometer. Additionally, its spatial resolution is dependent on the wavelength, which limits the acquisition of spatially resolved chemical distribution maps. In contrast, nanoscale near-field FTIR spectroscopy (nano-FTIR) can non-destructively characterize organic and inorganic compounds in meteorites with nanoscale spatial resolution (~ 20 nm) without the optical diffraction limit. Nano-FTIR spectroscopy is based on scattering-type scanning near-field optical microscopy (s-SNOM)^26^, where a metal coated conductive atomic force microscope (AFM) tip acts as an antenna for probing the molecular vibrations^27^. The tip can be illuminated by radiation of fixed wavelength (laser) or broadband (e.g., synchrotron) infrared radiation. In the latter case, FTIR spectra can be collected for a spatial resolution that is roughly equal to the radius apex of the probing tip^26–29^. Furthermore, the nano-FTIR spectroscopic imaging is independent of the wavelength. As a result of this independence, it can achieve a spatial resolution of ~ 20 nm^27,28,30–32^. Furthermore, nano-FTIR spectroscopy does not require powdered or chemically processed samples, and it does not consume the studied sample. The samples remain completely unaltered, and the petrographic context is fully retained. Therefore, nano-FTIR spectroscopy is a powerful analytical tool for the investigation of a variety of samples including meteoritic constituents. The mineralogies of a carbonaceous chondrite and a cometary dust grain have been investigated at ~ 20 nm spatial resolution using nano-FTIR, although within a limited (1100–800 cm^−1^) spectral range^33^. Recently, molecular variations at the ~ 20 nm spatial scale were shown for meteoritic minerals within 1600–850 cm^−1^^34^. Kebukawa et al.^35^ reported nano-FTIR analyses of organics and minerals in two carbonaceous chondrites with ~ 30 nm spatial resolution using photothermal nano-FTIR spectroscopy. To our knowledge, there is no other prior study that investigated chondritic organic matter using nano-FTIR spectroscopy.

In this study, we report the detection of C=O functional group organic compounds in situ in DOM 08006 (a CO3.0 chondrite) with ~ 20 nm spatial resolution using nano-FTIR spectroscopy. We interpret those compounds to be carbonyl compounds, specifically aldehydes and carboxylic acids. Petrographic context and topographic information on aldehydes and carboxylic acids in a carbonaceous chondrite are also presented. Our results show that the superior spot size (~ 20 nm spatial resolution), retained petrographic context, wavelength-independent and non-destructive nature make nano-FTIR spectroscopy one of the most ideal techniques for the investigation of extraterrestrial samples and their organic content.

## Results

**Figure 1:**
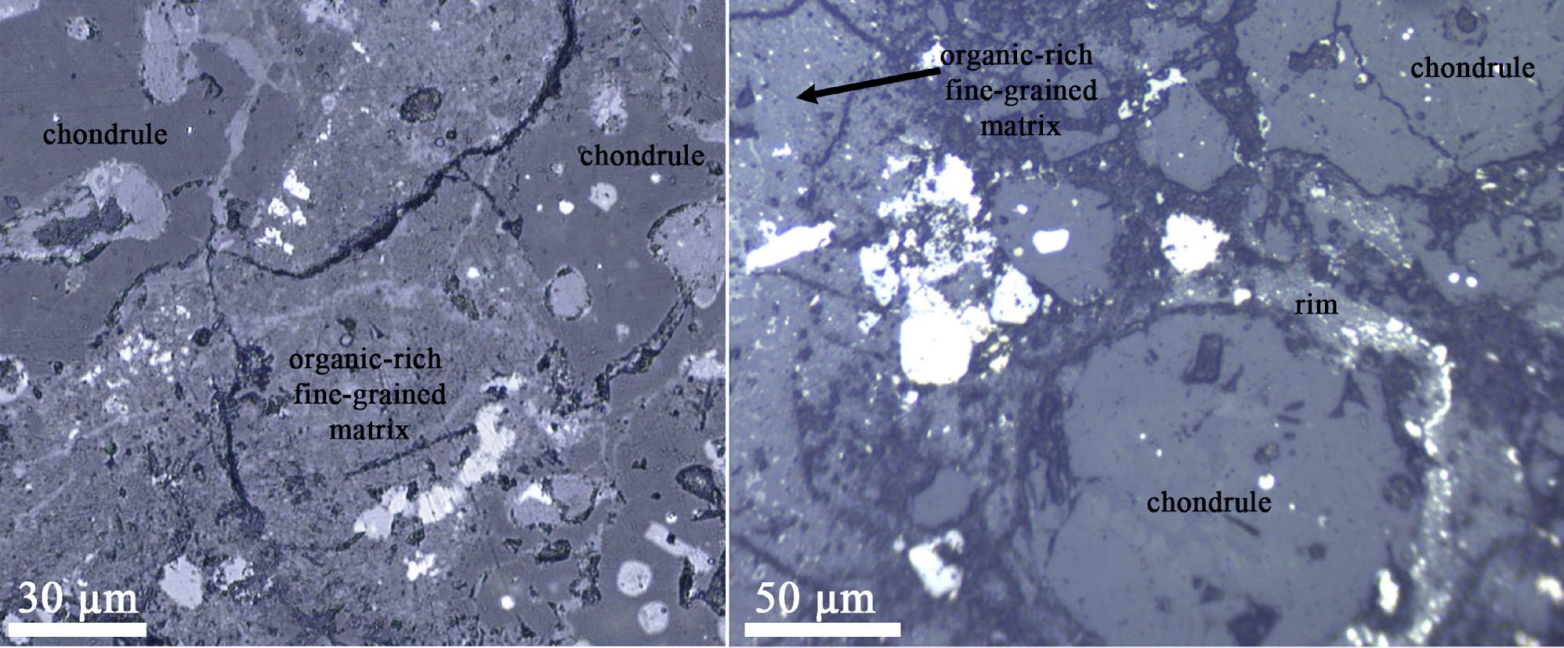
Reflected light visible micrographs of DOM 08006. The images show organic-rich fine-grained materials as well as rimmed chondrules. Opaque phases appear as bright objects.

**Figure 2:**
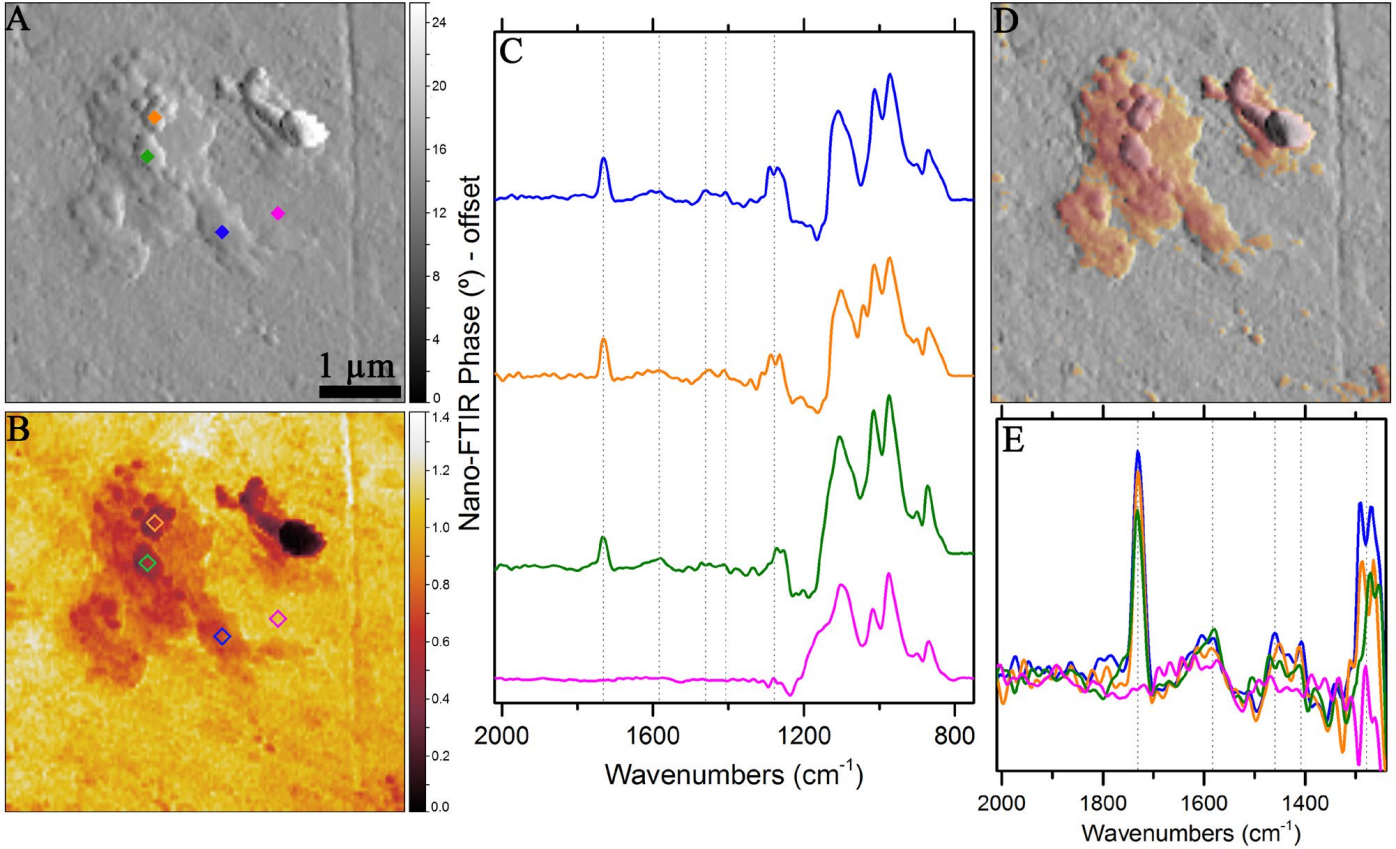
Nano-FTIR spectroscopic investigation of a location in DOM 08006. (**A**) Mechanical and (**B**) optical amplitude images of the measured area. Diamonds in (**A**) and (**B**) denote the points from where the spectra were collected. (**D**) The overlaid amplitude images indicating the boundaries and features of the organic compounds. (**C**) Corresponding color-coded nano-FTIR spectra. (**E**) Close up view of the organic peaks observed within 2000–1250 cm^−1^ in DOM 08006. Dashed lines indicate the positions of prominent organic features.

DOM 08006 has a fine-grained matrix material, rimmed chondrules, and various opaque phases (Fig. 1). Figure 2 shows nano-FTIR images and spectra of a location on DOM 08006. The mechanical amplitude image (Fig. 2A) shows ~ 1 × 2 µm^2^ organic material set within the matrix as positive relief features. Several roughly spherical and ~ 50–300 nm sized blobs of organic globules are observed on top of the matrix. The optical amplitude image (Fig. 2B), shows red (dark) and yellow (bright) colors corresponding to regions of higher and lower absorbance, respectively. The blended mechanical and optical amplitude images, as shown in Fig. 2D highlights the organic-rich regions. Figure 2C, E show the nano-FTIR spectra that were collected from the four points shown in the mechanical and optical amplitude images. Colors in the images correspond to the spectra. Nano-FTIR spectra present several infrared bands within the 1200–800 cm^−1^ range due to Si–O–Si stretching vibrations of silicate minerals. Blue, green, and orange spectra (collected from roughly spherical globules) present a prominent and sharp infrared band at 1730 cm^−1^. This band is attributed to C=O stretching vibrations^18,36,37^, likely in aldehyde. Moreover, the organic materials (blue, green, and orange diamonds in the images) also present a broad band centered near 1580 cm^−1^, also due to C=O stretching vibrations. This band can be attributed to carbonyls, specifically to carboxylic acids^38^. Carboxylic acids present another weak band near 1410 cm^−1^^39,40^, which is present as a sharp feature in the spectra of organic globules. The strong doublet at 1305–1250 cm^−1^ may be due to C–O stretching vibrational modes^41–43^. All these infrared bands and features are completely absent in the pink spectrum, which was collected from the matrix outside the organic material. Figure 2E presents a close-up version of the spectra within the 2000–1250 cm^−1^ region to clearly show the identified organic bands. Positive correlations between the organic peaks are clearly visible in Fig. 2E. The observed infrared bands, their positions, and possible assignments are presented in Table 1.

**Table 1:** Spectral positions and assignments of the organic matter and inorganic silicates observed via nano-FTIR spectroscopy.

Position (cm^−1^)	Position (µm)	Vibrational mode	Assignment
1730	5.78	C=O s	Carbonyls (aldehyde)
1580	6.33	C=O s	Carbonyls (carboxylic acid)
1458	6.85	C–H b	Aromatics
1408	7.10	C=O s	Carbonyls (carboxylic acid)
1440–1395	6.94–7.17	O–H b	Carbonyls (carboxylic acid)
1306–1245	7.65–8.03	C–O s	Aromatics
1160–850	8.62–11.76	Si–O s	Silicates

*s* stretching, *b* bending.

**Figure 3:**
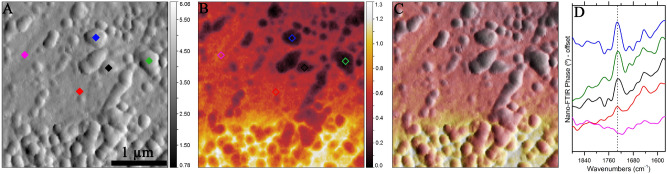
Nano-FTIR spectroscopic investigation of another location in DOM 08006. (**A**) Mechanical and (**B**) optical amplitude images of the measured area. Diamonds in (**A**) and (**B**) denote the points from where the spectra were collected. (**C**) Blended amplitude image clearly showing the roughly spherical carbonyl nanoglobules in DOM 08006. (**D**) Corresponding color-coded nano-FTIR spectra.

Figure 3A, B show, respectively, mechanical and optical amplitude images of another location on DOM 08006. This location consists of many roughly spherical 100–200 nm sized globules of organic material, set in a fine-grained matrix. Petrographic context and morphological/topological properties of the carbonyl compounds are clearly shown in the blended amplitude image, thanks to the superior spatial resolution of nano-FTIR spectroscopy (Fig. 3C). At this location, we focus on the 1880–1540 cm^−1^ spectral region, where the strongest C=O stretching vibrations occur (Fig. 3D). Out of the five nano-FTIR spectra collected from this location, three were from the spherical organic material and two from the matrix (Fig. 2C). Those from the organic material show a strong well-resolved band at 1730 cm^−1^ (indicated by a vertical dashed line in Fig. 2D) due to the C=O stretching modes. This band is absent in the spectrum collected from the matrix (pink), although the other matrix spectrum (red) shows a hint of this C=O stretching mode. This may have occurred as the red spectrum was collected right next to an organic particle that contributed to its spectrum. The lower part of the optical amplitude image in Fig. 2B shows a brighter matrix, indicating relatively high reflectivity. However, this is simply an artifact in the image.

**Figure 4:**
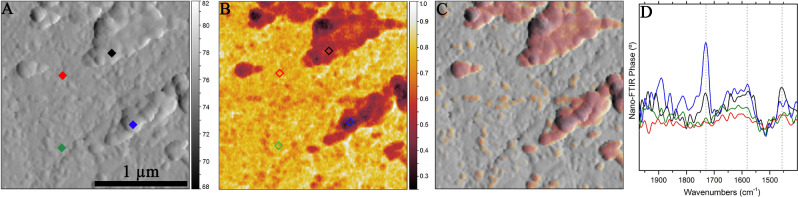
Nano-FTIR spectroscopic investigation of another location in DOM 08006. (**A**) Mechanical and (**B**) optical amplitude images of the measured area. Diamonds in (**A**) and (**B**) denote the points from where the spectra were collected. (**C**) Blended amplitude images indicating the boundaries and features of the organic compounds. (**D**) Comparison of the corresponding color-coded nano-FTIR spectra.

Similarly, Fig. 4 shows mechanical (Fig. 4A) and optical (Fig. 4B) amplitude images as well as nano-FTIR spectra (Fig. 4D) of the third location on DOM 08006, where a total of four nano-FTIR spectra were collected. While the matrix spectra do not present the C=O band at 1730 cm^−1^, it is present for the organic material. In fact, this band is the strongest in blue spectrum, which was collected from one of the darkest points in the optical amplitude image shown in Fig. 4B. The less dark organic region is indicated by the black diamond, which also shows the C=O band at a relatively low intensity. Similar to other areas discussed in the text above, the two spectra collected from the matrix do not present the C=O band. Moreover, the strength of the 1730 cm^−1^ band appears to be correlated with the band near 1580 cm^−1^, which is also attributed to C=O stretching modes in carbonyls, possibly carboxylic acids. Figure 4C highlights the locations, sizes, and boundaries of the identified carbonyl compounds. It is apparent that some of the organic blobs as small as ~ 20 nm are distributed over the matrix.

**Figure 5:**
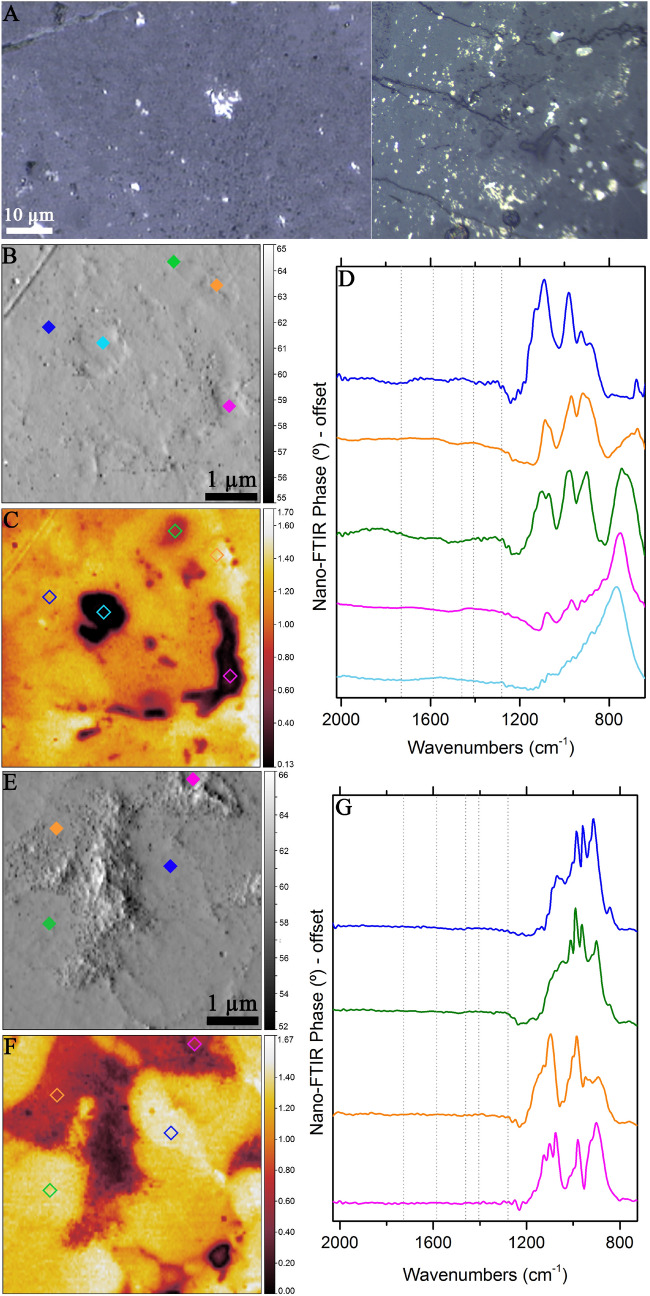
Nano-FTIR spectroscopic results of QUE 97344. (**A**) Visible micrograph of QUE 97344. (**B**, **E**) and (**C**, **F**) show, respectively, mechanical and optical amplitude images of the two locations on QUE 93744. Diamonds denote the points from where spectra were collected. (**D**, **G**). Corresponding color-coded nano-FTIR spectra, indicating presence of silicate bands and absence of the organic bands. Dotted vertical lines indicate positions of organics observed in DOM 08006.

In addition to DOM 08006, QUE 93744 (petrologic type 3 CV chondrite) was also measured using the same instruments and methods (Fig. 5A). Figure 5B, C, as well as 5E and 5F respectively present the mechanical and optical images of the studied locations. In our nano-FTIR investigations, no spherical organic globules were observed on QUE 93744. Nano-FTIR spectra of QUE 93744 do not reveal any of the C=O vibrational modes (Fig. 5D, G). The 1200–650 cm^−1^ region presents Si–O–Si stretching vibrations due to silicate minerals. Beyond 1200 cm^−1^, no sharp infrared features due to organics were observed. The absence of organics in QUE 93744, especially carbonyls similar to DOM 08006, could be attributed to several reasons. CO and CV chondrites may have formed from different starting materials and precursors. They may have also formed under different nebular conditions. Post-accretionary processes may also be responsible for the absence of carbonyls in QUE 93744. For instance, the peak metamorphic temperature of QUE 93744 was reported to be ~ 430–490 °C, twice as high as that for DOM 08006^44^. Such elevated temperatures might alternatively be responsible for the absence of carbonyls in QUE 93744.

## Discussion

The carbonyl group is an organic group in which carbon is double-bonded to oxygen. Calvin and Vaughn^45^ were some of the first to report carbonyls in meteorites. They reported the presence of a broad band at 1850–1650 cm^−1^ (attributed to carbonyls) in the infrared transmission spectra of organic matter that was chemically extracted from the Murray meteorite (CM2). Using mass spectroscopy, Hayes and Biemann^46^ studied the portion of the Murray meteorite that was thermally extracted from the larger host chip and vaporized it in a controlled chamber. Their results showed the presence of trace amounts of carbonyls in the meteorite. Hot water extracts of the Murchison meteorite (CM2) were investigated by Junglaus et al.^47^ using gas chromatography and mass spectroscopy. They reported the presence of low molecular weight aldehydes and ketones. More recently, Pizzarello et al.^48^ reported the presence of carbonyls in a solvent-extracted sample of the Tagish Lake (ungrouped C2) meteorite. A small feature in the FTIR spectra of crushed Tagish Lake meteorite was attributed to ketones^18^. Simkus et al.^49^ recently reported the presence of a suite of aldehydes and ketones in the solvent-extracted Murchison (CM2) meteorite.

Members of the carbonyl group include aldehyde, ketone, carboxylic acid, ester, and amide. Depending on factors such as mass and bond angles, the C=O stretching vibrations of the carbonyl group can occur between 1900–1550 cm^−1^^36^. Therefore, the infrared band at 1730 cm^−1^ observed in DOM 08006 is attributed to the C=O stretching vibrational modes of carbonyl compounds. Carbonyl group compounds give rise to a variety of infrared bands. For instance, aldehyde presents an infrared peak at 1730 cm^−1^^37^. Ketone peaks appear below 1730 cm^−1^, such as at 1720–1640 cm^−1^^18,50^. Carboxylic acids generally present a peak near 1723 ± 12 cm^−1^^51^. Some saturated carboxylic acids present a peak near 1730–1700 cm^−1^, while aromatic carboxylic acids present a peak at 1710–1680 cm^−1^. Ester peaks appear near 1740 cm^−1^^52^ or 1730 cm^−1^^53^. The amide^−1^ peak can appear at 1680–1630 cm^−1^^54^, such as at 1650 cm^−1^^55^. The amide-2 band appears at even lower frequencies, at 1550–1520 cm^−1^^54^ and at 1490–1460 cm^−1^^55^. These vibrational modes can shift to lower or higher frequencies (± 5–10 cm^−1^) due to the conjugated or saturated nature of the molecule. Nano-FTIR spectra of DOM 08006 yield strong absorbance peaks due to carbonyls such as aldehyde and carboxylic acids. The C=O stretching vibrational mode is associated with a large change in the net dipole moment^18^. This suggests abundant aldehydes and carboxylic acids in DOM 08006.

The meteoritic organic matter may have contributed to the origin and evolution of life on Earth. Therefore, some of the meteoritic organic matter is of astrobiological importance. Oxidation of aldehyde can form carboxylic acids^56^ and aliphatic monocarboxylic acids^57^, organic material that is important for the origin of life on Earth^57^. Aldehydes and ketones can also be precursors of the biologically necessary organic materials in the solar system^57^, for instance oxidized aldehyde may be the building block of biological membranes^57,58^. On the other hand, the origin of carbonyl compounds in carbonaceous chondrites is still ambiguous. Low temperature aqueous alteration of insoluble^59^ as well as soluble organic matter can be the source of meteoritic carbonyl compounds. For instance, organic nanoglobules may form in a low temperature aqueous environment from formaldehyde, a type of aldehyde^60^. This is consistent with low petrologic type chondrites (such as types 1 and 2, which experienced thermal metamorphic temperatures of ≤ 150 °C^61^), evident from the fact that the majority of previous studies showed the presence of carbonyls in those types of chondrites. However, we have shown the presence of abundant carbonyls in DOM 08006, a slightly more heated petrologic type 3.0 CO chondrite whose Raman carbon geothermometry calculation points to peak metamorphic temperatures of ~ 220–230 °C^44^. This suggests a pathway through which aldehyde and carboxylic acids can form and remain intact under such elevated temperatures. This also suggests that the aqueous alteration processes experienced by the petrologic type 1 and 2 chondrites inherit, preserve, and/or form more carbonyl compounds that are initially present in type 3.0 chondrites.

Previously, the detection of carbonyl compounds in extraterrestrial materials was carried out using destructive methods in most studies. Typically, samples were demineralized and organic material was chemically extracted (^62^ and references therein). In addition to the loss and consumption of the extraterrestrial material, the petrographic context as well as morphological information on carbonyls were also lost. FTIR spectroscopy is completely non-destructive and can detect signatures of sufficiently large organic matter in standard meteorite sections. However, meteoritic organic matter is typically sub-micron in size and conventional FTIR spectroscopy is very likely to overlook such organics owing to its low overall abundance. We illustrate and confirm that high spatial resolution nano-FTIR spectroscopy can detect and measure organic material as small as ~ 20 nm present within the matrix of carbonaceous chondrites, owing to the preserved petrographic context noted via nano-FTIR spectroscopy.

## Methods

### Samples

DOM 08006 and QUE 93744 were obtained from the Astromaterials Acquisition and Curation Office at NASA Johnson Space Center. Both samples were cut dry using a band saw and the resultant ~ cm scale thick slab was glued on a glass slide using epoxy. The thick slabs were then further processed using diamond paste to make thin (~ 30–40 µm thickness) sections with flat and smooth surfaces. This kind of sample preparation and the use of epoxy resin may cause the contamination of the samples; however, as shown in the Discussion section (and in the Supplementary Information), this was not the case in this study.

### Nano-FTIR spectroscopy

Nano-FTIR spectroscopic experiments were conducted using a commercial s-SNOM nano-FTIR imaging system (neaspec GmbH) equipped with multiple mid-infrared broadband lasers. Spectra were collected with ~ 20 nm spatial and 12 cm^−1^ spectral resolution from selected areas/points on the surface of samples within the 2000–650 cm^−1^ spectral range. This region covers the major silicate peaks and organic functional groups. For each spectrum, 10 spectra were coadded (integration time was 10 ms/spectrum) to create the average spectrum. AFM scans were collected using a Pt coated neaspec cantilever tip (resonance frequency 250–270 kHz) that maps the surface topography. The reference spectra were recorded on a silicon wafer. In this setup, the Michelson interferometer generates amplitude and phase spectra simultaneously. These spectra represent reflection and absorption spectra, respectively. In this study, we report the phase spectra as these can be interpreted as the infrared absorbance. In addition to nano-FTIR amplitude and phase spectra, each measurement also generates mechanical and optical amplitude images. The mechanical amplitude is related to the topography of the sample, and represents the height of the AFM tip relative to the sample surface. The optical amplitude images, which are associated with the reflectivity of the sample surface, are measured with the nano-FTIR interferometer at a fixed delay position where all frequencies produce constructive interference. This interferometer position, also called white light position, produces the maximum infrared reflectivity signal. Thus, the optical amplitude images, which are the integrated spectral signal generated in this measurement mode, represent broadband reflectivity integrated across the full wavelength range of the utilized laser. Reproducibility of the spectra was ensured by measuring the same point twice in each region of interest, one at the beginning and one at the end of the spectral measurement series. Sample drift was also monitored constantly by checking the reference points on the sample. This drift correction ensures that the uncertainty in the position of the measured point is minimal, ideally less than the spatial resolution during the collection of each spectrum.

## Supplementary Information

**Supplementary Information.**
