# Supplementary material for: Nano-FTIR spectroscopic identification of prebiotic carbonyl compounds in Dominion Range 08006 carbonaceous chondrite
Source: Sci Rep. 2021 Jun 2;11:11656. doi: 10.1038/s41598-021-91200-8 (PMC8172632; doi:10.1038/s41598-021-91200-8)
Supplement: Supplementary file 1 — Supplementary Information. [file 41598_2021_91200_MOESM1_ESM.pdf]

Supplementary Information

Nano-FTIR spectroscopic identification of prebiotic carbonyl compounds in Dominion Range 08006 carbonaceous chondrite

Mehmet Yesiltas*, Timothy D. Glotch, and Bogdan Sava

* Corresponding author: myesiltas@knights.ucf.edu

Supplementary Information Text

Carbonaceous chondrites are highly susceptible to contamination. Even storage materials and containers can contaminate meteorites within a day [1]. Therefore, extra caution must be taken when investigating organic materials within meteorites. In our case, the studied meteorite samples were not stored in materials with the ability to outgas and contaminate samples. A piece of the sample was cut and subsequently prepared in the form of a thin section. The spectral evidence of contamination is the occurrence of a weak and sharp Si-CH3 band at 1260 cm^{-1} in the infrared spectra due to outgassing silicon and/or rubber materials [1]. It the sample were contaminated, this infrared feature should be present in all spectra collected within the same location in the meteorite. However, this is not the case. Spectra of DOM 08006 present a doublet at 1310–1230 cm^{-1} due to C-O stretching vibrations. The correlation between the intensity of this doublet and that of other carbonyl peaks (see Fig. 2E) indicates that C-H contamination does not contribute to the doublet. In addition, the spectrum of the matrix (which is presumably phyllosilicate-rich) in DOM 08006 does not show any feature near 1260 cm^{-1} (e.g., pink spectrum in Fig. 2C). None of the spectra collected from QUE 93744 exhibit a feature at 1260 cm^{-1} either. Moreover, in addition to the 1260 cm^{-1} peak, the presence of Si-CH3 also gives rise to a peak at ~780 cm^{-1} [2]. As seen in Fig. 2C, this feature is absent in all the spectra. As a result, C-H contamination of the investigated samples in the laboratory seems unlikely.

Sample preparation procedures can potentially contaminate the meteorites. If this is the case, all meteorites prepared in the same way should all be contaminated. However, we do not see this in the current study. Namely, QUE 93744 was prepared the same way as DOM 08006, and the collected nano-FTIR spectra do not show infrared features of contaminant organic matter.

Another possible source for contamination could be the epoxy resin used during the sample preparation. To check this, we collected nano-FTIR spectra and AFM images of epoxy resin. Fig. S1 shows mechanical and optical amplitude images as well as the corresponding color-coded nano-FTIR spectra of epoxy on DOM 08006. As seen in Fig. S1C, peak positions and spectral profiles of epoxy are quite different from the meteorite data. The epoxy infrared peaks appear at 1509, 1245, 1039, and 832 cm^{-1}. None of these bands are present in our meteorite spectra. In addition, the epoxy spectra do not present a peak near 1730 cm^{-1} (indicated by a vertical dashed line in Fig. S1C). Therefore, we believe the presented spectra are free of contamination and that the organic compounds observed in DOM 08006 are indigenous.

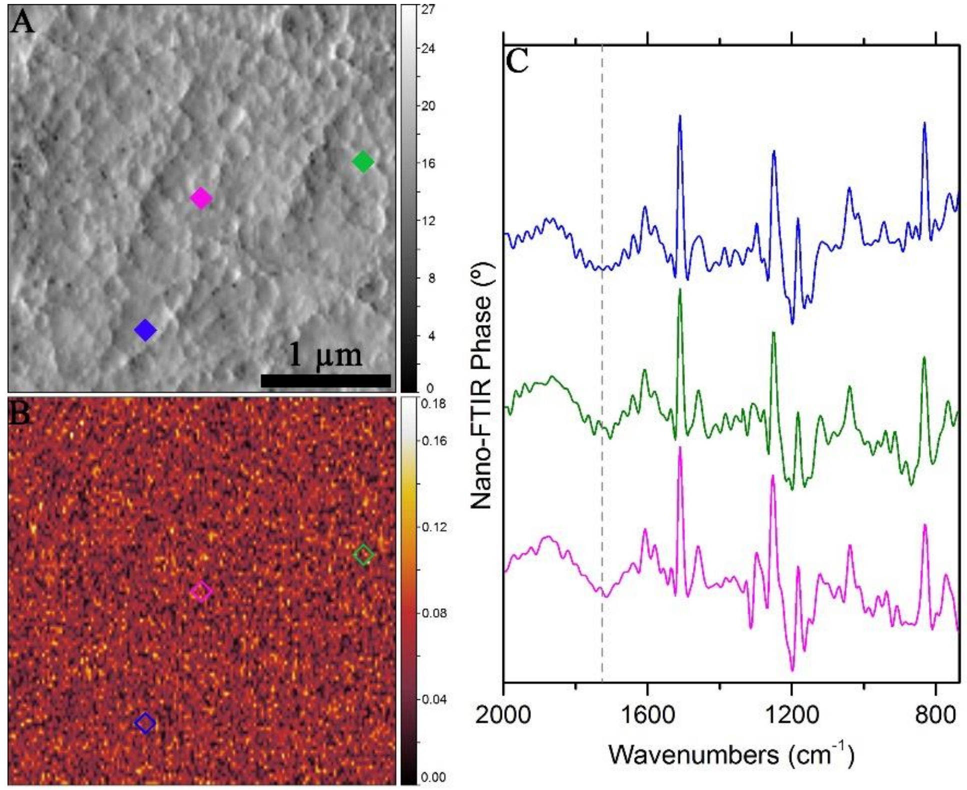

Fig. S1. Mechanical (A) and optical (B) amplitude images of epoxy. Diamonds denote the points where spectra were collected. Corresponding color-coded nano-FTIR spectra are shown in (C).

SI References

1. Y. Kebukawa, et al. (2009) Rapid contamination during storage of carbonaceous chondrites prepared for micro FTIR measurements. *Meteoritics & Planetary Science* 44(4):545-557.

2. L.M. Johnson, et al. (2013) Elastomeric microparticles for acoustic mediated bioseparations. *Journal of nanobiotechnology* 11(1):1-8.
